# Circulating neutralizing antibodies and SARS-CoV-2 variant replication following postvaccination infections

**DOI:** 10.1172/jci.insight.185953

**Published:** 2025-03-10

**Authors:** Miguel A. Garcia-Knight, J. Daniel Kelly, Scott Lu, Michel Tassetto, Sarah A. Goldberg, Amethyst Zhang, Jesus Pineda-Ramirez, Khamal Anglin, Michelle C. Davidson, Jessica Y. Chen, Maya Fortes-Cobby, Sara Park, Ana Martinez, Matthew So, Aidan Donovan, Badri Viswanathan, Eugene T. Richardson, David R. McIlwain, Brice Gaudilliere, Rachel L. Rutishauser, Ahmed Chenna, Christos Petropoulos, Terri Wrin, Steven G. Deeks, Glen R. Abedi, Sharon Saydah, Jeffrey N. Martin, Melissa Briggs Hagen, Claire M. Midgley, Michael J. Peluso, Raul Andino

**Affiliations:** 1 Department of Immunology and Microbiology, UCSF, San Francisco, California, USA.; 2 Departamento de Inmunología, Instituto de Investigaciones Biomédicas, Universidad Nacional Autónoma de México, Mexico City, Mexico.; 3 Department of Medicine,; 4 Department of Epidemiology and Biostatistics,; 5 Institute for Global Health Sciences, and; 6 F.I. Proctor Foundation, UCSF, San Francisco, California, USA.; 7 San Francisco VA Medical Center, San Francisco, California, USA.; 8 Department of Medicine, Brigham and Women’s Hospital, Boston, Massachusetts, USA.; 9 Department of Global Health and Social Medicine, Harvard Medical School, Boston, Massachusetts, USA.; 10 Department of Microbiology and Immunology and; 11 Department of Anesthesiology, Perioperative and Pain Medicine, Stanford University, Stanford, California, USA.; 12 Division of Experimental Medicine, Department of Medicine, UCSF, San Francisco, California, USA.; 13 Labcorp - Monogram Biosciences, South San Francisco, California, USA.; 14 Division of HIV, Infectious Diseases and Global Medicine, Zuckerberg San Francisco General Hospital, San Francisco, California, USA.; 15 Coronavirus and Other Respiratory Viruses Division, National Center for Immunization and Respiratory Diseases, Centers for Disease Control and Prevention, Atlanta, Georgia, USA.

**Keywords:** COVID-19, Immunology, Infectious disease, Adaptive immunity

## Abstract

The effect of preexisting neutralizing antibodies (NAb) on SARS-CoV-2 shedding in postvaccination infection (PVI) is not well understood. We characterized viral shedding longitudinally in nasal specimens in relation to baseline (pre/periinfection) serum NAb titers in 125 participants infected with SARS-CoV-2 variants. Among 68 vaccinated participants, we quantified the effect of baseline NAb titers on maximum viral RNA titers and infectivity duration. Baseline NAbs were higher and targeted a broader range of variants in participants with monovalent ancestral booster vaccinations compared with those with a primary vaccine series. In Delta infections, baseline NAb titers targeting Delta or Wuhan-Hu-1 correlated negatively with maximum viral RNA. Per log_10_ increase in Delta-targeting baseline NAb IC_50_, maximum viral load was reduced –2.43 (95% CI: –3.76, –1.11) log_10_ nucleocapsid copies, and infectious viral shedding was reduced –2.79 (95% CI: –4.99, –0.60) days. Conversely, in Omicron infections (BA.1, BA.2, BA.4, or BA.5), baseline NAb titers against Omicron lineages or Wuhan-Hu-1 did not predict viral outcomes. Our results provide robust estimates of the effect of baseline NAbs on the magnitude and duration of nasal viral replication after PVI (albeit with an unclear effect on transmission) and show how immune escape variants efficiently evade these modulating effects.

## Abstract 2

Higher neutralizing antibody titres against SARS-CoV-2 before an infection limits virus replication and infection duration, however escape variants evade this effect.

## Graphical abstract

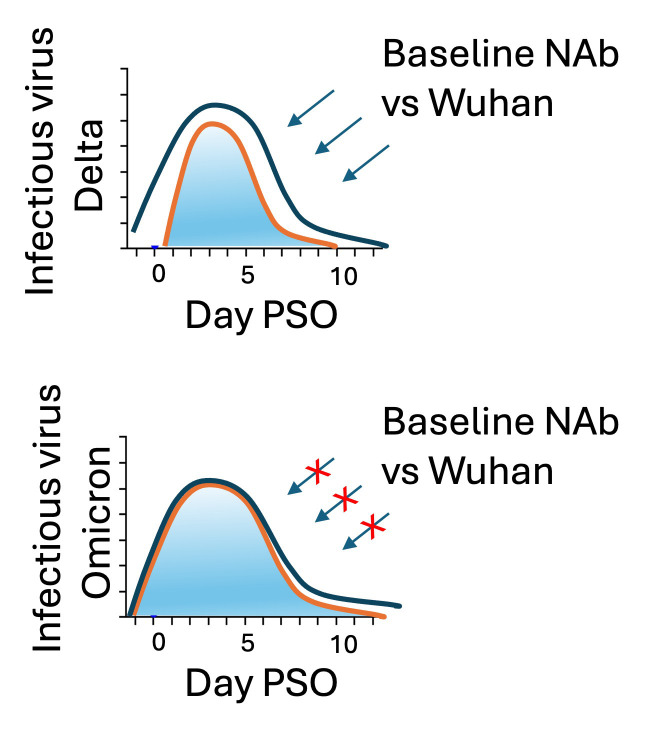

## Introduction

Circulating neutralizing antibodies (NAbs) against SARS-CoV-2 are associated with protection against infection and disease and are induced following both SARS-CoV-2 infections and vaccination (1). However, NAbs titers wane in the months following their induction (through vaccination or infection), and time since vaccination correlates negatively with protection (2, 3). In addition, variants such as those descending from the B.1.1.529 (Omicron) lineage (with > 30 amino acid mutations in the spike protein relative to Wuhan-Hu-1) can evade NAbs targeting ancestral lineages and vaccine antigens (4). This has contributed to widespread postvaccination infections (PVI), reinfections, and ongoing waves of community transmission, despite the use of distinct vaccine platforms and the provision of booster vaccinations with updated vaccine antigens (5). However, most vaccine doses received globally, either as part of a primary vaccine series or through booster doses, have used antigens derived from ancestral Wuhan-Hu-1 (6).

Numerous studies have assessed the effect of vaccination on viral replication dynamics and infectiousness — key parameters linked to SARS-CoV-2 pathogenesis and transmission. However, few studies have been able to directly assess how these outcomes relate to the NAb response. For instance, the mRNA vaccine BNT162b2 can affect peak viral load in PVI early after vaccination (7), though the effect is transient and not observed in all studies of outpatient cohorts (8, 9). Likewise, data on the effect of mRNA vaccines on the duration of viral shedding and infectiousness following PVI are conflicting (10); there is no reduction in the incidence of household transmission in Delta infections (9, 11), particularly 12 weeks after vaccination (12). In the present study, we determine the relationship between NAb titers — elicited following vaccination and measured at the time of infection — and key virological parameters in a longitudinal household cohort sampled intensely during the period of viremia. Our findings help quantify the protective effect of circulating NAbs against SARS-CoV-2 variants with implications for the study of COVID-19 pathogenesis and for efforts to model SARS-CoV-2 transmission in the context of novel prophylactic and therapeutic interventions.

## Results

### Cohort characteristics.

A total of 174 participants (125 SARS-CoV-2–infected and 49 uninfected) from 78 households were enrolled and had blood collected from September 2020 through September 2022 in San Francisco, California, USA. A median of 13 (range, 3–15) nasal specimens and 4 (range, 1–4) blood specimens were collected per participant, totaling 2,471 and 664 specimens, respectively. The demographic characteristics and vaccination histories of infected and uninfected participants are shown in Supplemental Table 1 (supplemental material available online with this article; https://doi.org/10.1172/jci.insight.185953DS1). Participants were infected with distinct variants including those that predated the emergence of variants of concern (preVOC), Epsilon, Delta, and Omicron (sublineages BA.1, BA,2, BA.4, or BA.5). Among infected participants, 37 of 125 (29.6%) received a primary vaccine series and 31 of 125 (24.8%) received 1 or 2 original monovalent booster doses. A total of 45 of 57 (85%) unvaccinated participants were infected with preVOC viral lineages, 32 of 37 (87%) of those who received a primary vaccine series were infected with Delta variants, and 31 of 31 (100%) participants who received original monovalent booster vaccinations had infections with Omicron variants.

### Viral shedding kinetics over the acute infection period.

**Figure 1:**
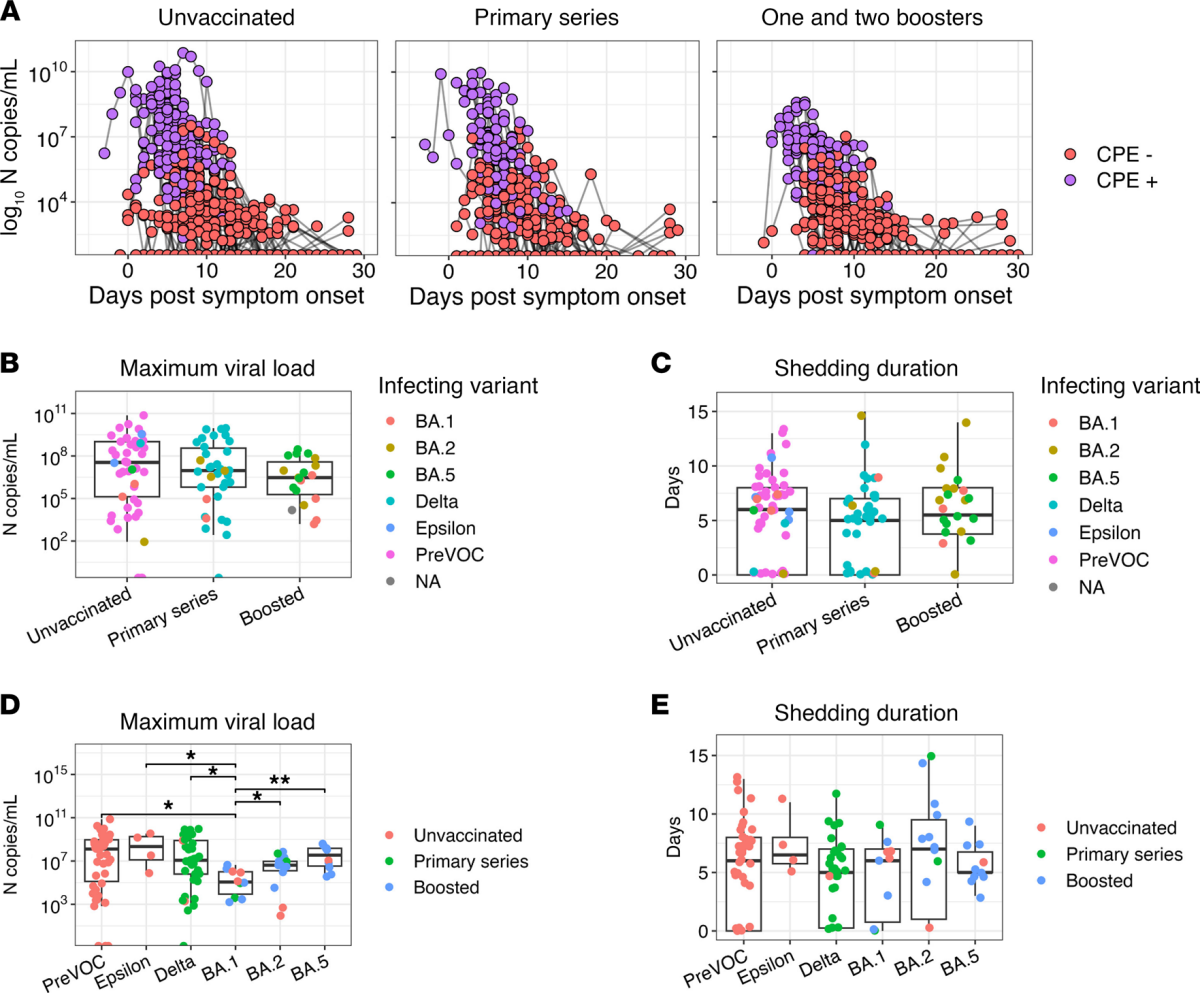
The influence of vaccination history and viral variant on viral shedding dynamics. (**A**) Longitudinal viral shedding dynamics in nasal specimens collected over 28 days from symptom onset in unvaccinated participants (*n* = 57) and participants with postvaccination infections (PVI) who received a primary vaccine series (*n* = 37) or monovalent booster vaccinations (*n* = 31). Copies of SARS-CoV-2 nucleocapsid (N) RNA of each specimen and the presence of infectious virus are shown. (**B**) Comparison of maximum copies of N RNA between vaccine groups. (**C**) Comparison of the duration in days in which infectious virus was detected between vaccine groups. (**D**) Comparison of maximum copies of N RNA in participants stratified by infecting variant. Vaccination history is indicated by the color shown in the legend. (**E**) Comparison of duration in days in which infectious virus was detected stratified by infecting variant. Vaccination history is indicated the color shown in the legend. Box plots represent the IQR (box), median (line), and IQR × 1.5 values (whiskers). All pairwise comparisons were made using a 2-sided Wilcoxon rank sum test. Only statistically significant differences are shown. *P < 0.05 and **P < 0.01. CPE, cytopathic effect.

Among infected participants, we quantified viral RNA and assessed the presence of infectious virus longitudinally, by vaccine status (Figure 1A). Maximum viral RNA copies and the duration of infectious virus shedding did not differ significantly by vaccine status (Figure 1, B and C). Analysis of kinetics by variant indicated that participants with BA.1 infections had a significantly reduced maximum RNA load than those with pre-BA.1 infections or those with BA.2/BA.4 and BA.5 infections (Figure 1D). No differences were observed between variants with regard to the duration of infectious virus shedding (Figure 1E).

### Baseline neutralizing antibody titers in participants with PVIs.

Recruitment specimens were collected a median of 5 (interquartile range [IQR]: 4–5) days postsymptom onset (PSO) in individuals infected. To focus on NAb titers prior to the induction of the anamnestic response following PVI, we excluded from our analysis participants (*n* = 22) with recruitment specimens obtained on day 7 PSO or later (13). No significant differences were observed in median NAb titers in recruitment specimens obtained < 7 days PSO between participants with PVI and uninfected participants stratified by vaccination status (Supplemental Figure 1), indicating that baseline titers < 7 days after onset in participants with infections that resembled those prior to infection rather than reflecting postinfection responses. In addition, there was no difference in the time since last vaccine dose between PVI groups (Supplemental Figure 2)

**Figure 2:**
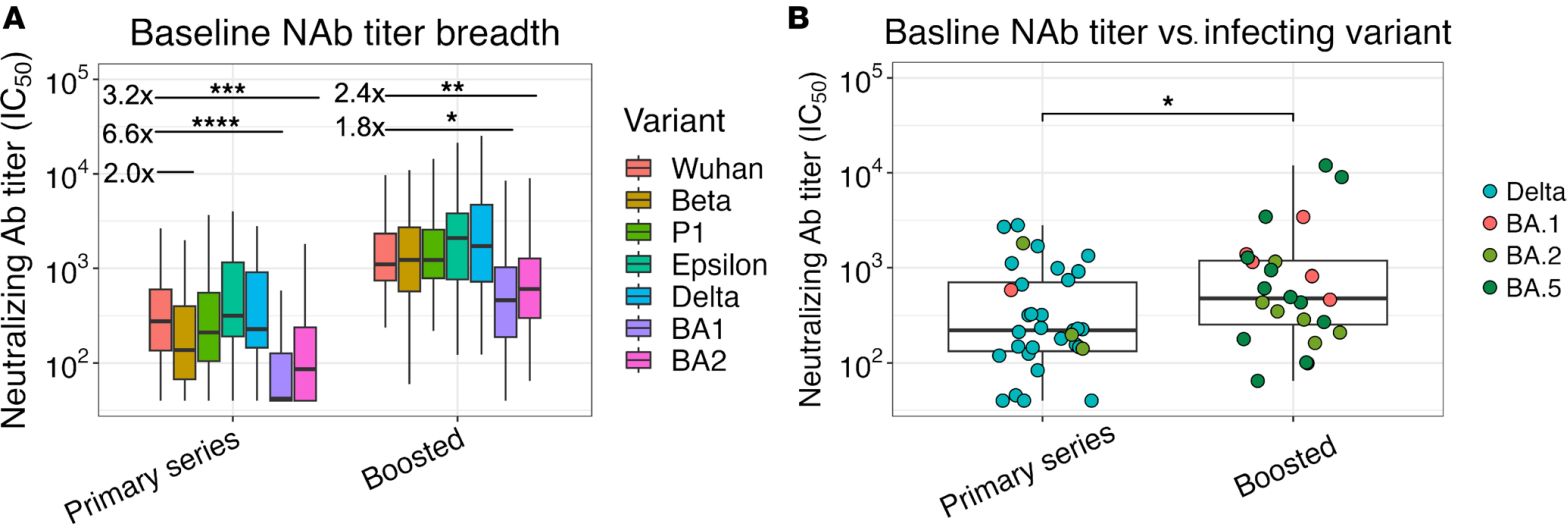
The magnitude and breadth of baseline NAb titers in participants with PVI. (**A**) NAb titers targeting SARS-CoV-2 variants compared with Wuhan-Hu-1 in participants with PVI who received a primary vaccine series or monovalent booster vaccination. *n* = 22 participants with recruitment specimens taken > 7 PSO were excluded from the analysis. (**B**) Comparison of baseline NAb titers targeting the infecting variant (except for BA.5 infections for which responses against BA.2 are shown) between participants with PVIs who received a primary vaccine series or monovalent booster vaccination. Box plots represent the IQR (box), median (line), and IQR × 1.5 values (whiskers). Statistical comparisons were made using a 2-sided Wilcoxon rank sum test. Comparisons with *P* < 0.1 are shown. *P < 0.05, **P < 0.01, ***P < 0.001, and ****P < 0.0005.

We initially assessed the strength and breadth of the baseline NAb response (Figure 2A). We observed that, in participants with a primary vaccine series, titers against Omicron BA.1 and BA.2 were reduced 6.6-fold (*P* > 0.0001) and 3.2-fold (*P* < 0.001), respectively, compared with Wuhan-Hu-1 and tended to be reduced 2.0-fold (*P* = 0.08) against Beta. By contrast, in participants who received booster vaccinations, no reduction in baseline NAb titers was observed against Beta, and NAb titers were only reduced 2.4-fold (*P* = 0.005) and 1.8-fold (*P* = 0.001) against Omicron BA.1 and BA.2, respectively (Figure 2A). Overall, 17 of 37 (46%) and 10 of 37 (27%) participants with a primary vaccine series had undetectable NAb responses to BA.1 and BA.2, respectively, compared with 1 of 31 (3%) and 0 of 31 (0%) participants who received booster vaccinations. Baseline NAb titers targeting the infecting variant for each participant (except those with BA.5 infections for which response against BA.2 are shown) were higher in those who had received vaccine booster doses compared with those who received a primary series (*P* < 0.05) (Figure 2B).

### Viral replication and duration of infection following PVIs are associated with baseline NAb titers in a variant-specific manner.

**Figure 3:**
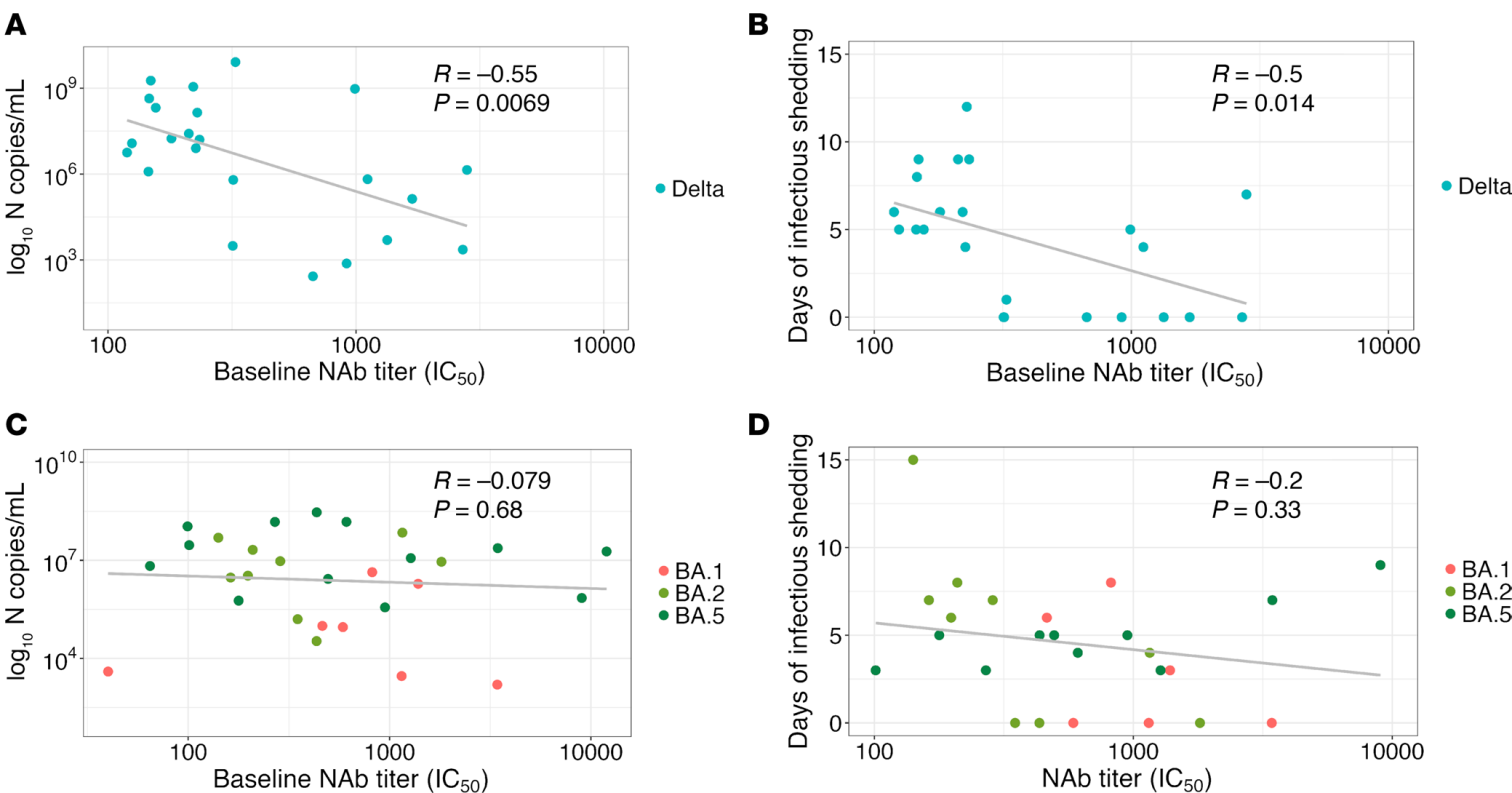
The relationship between baseline NAb titers and viral shedding outcomes. (**A** and **B**) The correlation between baseline NAb titer targeting the infecting variant in vaccinated participants (*n* = 29) infected with Delta variants and maximum RNA viral copies (**A**) and infectious virus shedding (**B**) over the study period. (**C** and **D**) The correlation between baseline NAb titer targeting the infecting variant (except for participants with BA.5 infections for which responses were against BA.2) in vaccinated participants (*n* = 29) infected with Omicron variants and maximum RNA viral copies (**C**) and infectious virus shedding (**D**) over the study period. Participants (*n* = 9) with recruitment specimens taken on day 7 PSO or later were excluded. Pearson’s correlation coefficients and infecting variant are shown.

Given that the strength of the baseline NAb titers differed by targeted variant, we next assessed the correlation between baseline NAb titers against the infecting variant (or BA.2 in the case of BA.5 infections) and features of viral replication dynamics (Figure 3). In participants infected with Delta variants, we observed a significant negative correlation between baseline Delta-specific NAb titers and both maximum viral RNA load (*R* = –0.55, *P* < 0.0069; Figure 3A) and the duration of infectious virus shedding (*R* = –0.5, *P* = 0.014; Figure 3B). A negative correlation was also observed between baseline NAb titers targeting Wuhan-Hu-1 and both maximum viral RNA load (*R* = –0.43, *P* = 0.048; Supplemental Figure 3A) and the duration of infectious virus shedding (*R* = –0.53, *P* = 0.012; Supplemental Figure 3B). In participants infected with Omicron variants, baseline titers against the infecting variant (Figure 3, C and D) or against Wuhan-Hu-1 (Supplemental Figure 3, C and D) were not associated with either viral load or duration of infectious viral shedding.

To further quantify the effect of baseline NAbs on virological outcomes following PVI (maximum viral load and duration of infectious shedding), we used multivariable linear regression in separate models for participants with Delta and Omicron infections. We adjusted for age and time since last vaccination (in Delta and Omicron infections) and for Omicron variant (BA.1 BA.2 or BA.5) in Omicron infections (Table 1). We did not adjust for vaccine status in our 2 models, as vaccination status was colinear with variant infection in our cohort. Higher baseline NAb titers were independently associated with lower peak viral load and shorter duration of infectious viral shedding in Delta infections; per log_10_ increase in baseline NAb titer, we observed a –2.43 reduction in log_10_ maximum viral load (95% CI: –3.76, –1.11; *P* = 0.0009; Table 1), and a –2.79 day reduction in duration of infectious virus shedding (95% CI: –4.99, –0.60; *P* = 0.02; Table 1). However, in Omicron infections, no significant associations were observed between baseline NAb titers and maximum viral RNA load or the duration of infectious virus shedding (Table 1). In line with univariate results in Figure 1D, BA.1 infection was independently associated with reduced maximum viral RNA titers compared with BA.2/4 (*P* = 0.01) and BA.5 infections (*P* = 0.004).

## Discussion

Our findings show that, following PVI (in people vaccinated with ancestral spike antigens), higher baseline NAb titers are associated with accelerated viral clearance dynamics following infections with Delta variants, and we provide robust estimations to help quantify this effect. In addition, we find that, in participants with PVI, baseline NAb titers targeting a range of variants up to BA.2 were increased in those who received booster vaccine doses compared with a primary series alone, as previously reported (14, 15), and that booster vaccination was associated with a greater breadth of response including robust responses against early circulating immune escape variants Beta and P1. However, baseline NAb titers targeting BA.1 and BA.2 variants were significantly reduced compared with ancestral variants in all vaccinated participants, and viral clearance was not influenced by NAb titers in participants infected with Omicron variants. This suggests that NAbs generated through first-generation vaccines are limited in their ability to target conserved epitopes in the spike protein of Omicron variants and support the use of booster vaccination with updated antigens that may further broaden the NAb response.

By quantifying the effect of baseline NAbs on viral clearance, our findings may help parameterize mathematical models of transmission dynamics (16, 17) and immunobridging studies for the development of novel vaccines (18); they may also help define the correlates of protection against SARS-CoV-2 transmission and disease. To this end, further assessments of the relationship between circulating NAbs, mucosal NAbs at the site of infection, and viral shedding dynamics are warranted. While our data implicate serum NAb in viral control, mucosal spike-binding IgG and, to a lesser extent, IgA have been found to correlate with the serum responses early after infection (19). In addition, binding mucosal IgA levels have been associated with reduced risk of PVI with Omicron in individuals with previous infections (20) and with viral RNA clearance dynamics (21), and dimeric IgA found in mucosa has been shown to have potent neutralizing activity (22, 23). However, although IgA likely contributes to the prevention of viral replication in the upper respiratory tract, its induction and maintenance is associated mainly with SARS-CoV-2 infection rather than repeated systemic vaccination (21, 24, 25). Since our study largely focused on participants with primary infections with immunity primed through vaccination, particularly those with Delta PVI, our data suggest that mucosal IgG may have played an important role in modulating the nasal viral replication dynamics we observed. In addition, recall serum CD4 and CD8 T cell responses, elicited through vaccination, have been associated with the control of viral replication following PVI and with protective immunity (26, 27). Several studies have also shown that SARS-CoV-2–specific T cell responses are preserved against VOCs such as Omicron that successfully evade NAb responses (28, 29), contributing to the breadth of immunity and potentially viral control in the absence of an effective NAb response. T cells are also critical for the maintenance of a coordinated immune responses against SARS-CoV-2 that may contribute to limiting systemic pathology associated with COVID-19 (30). As vaccine strategies that boost respiratory mucosal immunity emerge (31, 32), it will be important to assess that role of the maintenance of systemic immunity (humoral and cell mediated) and protection from SARS-CoV-2 pathology in both respiratory and nonrespiratory tissues.

One of the strengths of our study is the accurate estimation of maximum RNA titers and duration of shedding of infectious virus through analysis of nasal specimens collected daily from participants. Assessments of the effect of vaccination status on viral shedding dynamics have reported contrasting results, with initial studies suggesting an effect on peak RNA viral loads compared with unvaccinated individuals following infection with Alpha variants (33). However, reports of Delta and Omicron BA.1 infections in outpatient cohorts (8, 9, 34) and in individuals 2–6 months after receiving the last vaccine dose (7), suggest no substantial effect on viral RNA load. Accordingly, our analysis including unvaccinated individuals, indicated no association between vaccination status and the virological outcomes included in our study. Taken together, this suggests that any positive effects of first-generation vaccines on viral shedding outcomes and, consequently, on transmission may be rapidly lost (within 6 months following the last vaccine dose). In contrast to the effect of vaccination, infection with Omicron BA.1 was associated, in both univariate and multivariable analyses, with reduced maximum RNA titers compared with pre-Delta, Delta, and BA.5 infection, as previously suggested (35). The mechanisms by which BA.1 remained infectious at low viral loads (36), achieving attack rates that exceeded those of other variants (37) with lower peak viral titers, remains to be fully understood, although there is evidence that mutations accumulated in spike reduce the barrier to infection through accelerated early-infection kinetics and evasion of innate immunity compared with Delta and pre-Delta variants (38, 39).

Our study has some limitations. We were not able to measure baseline NAb titers to BA.5 or to currently circulating Omicron variants, underscoring the importance of continued monitoring of the strength and breadth of immune responses against emergent antigenically divergent lineages such as JN.1 and KP.3. We used BA.2 as a proxy to estimate NAb responses to BA.5 in those who had BA.5 infections. Given that BA.5 descended from BA.2 and that all vaccinations contained ancestral spike antigens, we expect the immune escape phenotype of BA.5 to affect the results in Figure 3, C and D, and Table 1 in similar manner to BA.2. In addition, we were not able evaluate the effect of vaccine boosters containing updated spike antigens, such as those containing sequences from BA.4/5 or XBB.1.5. A recent study showed equivalent NAbs responses against distinct Omicron lineages after a fourth-dose booster with either a BA.4/5-containing bivalent or an ancestral monovalent booster, suggestive of immunological imprinting (40). By contrast, broadening of the NAb response following BA.4/5-containing bivalent booster or monovalent XBB.1.5 booster with robust targeting of XBB1.5 and JN.1, respectively, has also been reported (41, 42), potentially contributing to increased vaccine effectiveness (43). It remains to be seen whether enhancing baseline neutralizing antibody (NAb) titers through updated vaccine antigens can reestablish the correlation with viral shedding outcomes in primary viral infections (PVI) involving current circulating variants. Similarly, further research is needed to better define the antigenic distance (41, 44) between vaccine antigens and infecting variants at which the relationship between baseline NAb titers and viral clearance dynamics is disrupted.

## Methods

### Sex as a biological variable.

Our study examined male and female participants, and similar findings are reported for both sexes.

### Study population and design.

This observational longitudinal cohort, recruited in San Francisco, California, USA, was designed to characterize virological, immunological, and clinical outcomes of SARS-CoV-2 infection and household transmission dynamics, as previously detailed (11). Briefly, both index cases and household contacts were recruited if an index case was identified from individuals with a positive health provider–ordered SARS-CoV-2 nucleic acid amplification test result on a nasopharyngeal or oropharyngeal (NP/OP) specimen done at UCSF-affiliated health facilities. Index cases were defined as being infected with SARS-CoV-2 within 5 days of symptom onset by clinical nucleic acid amplification tests at study health facilities. Household contacts were defined as cohabitants of the index participant who did not report COVID-19–like symptoms in the preceding week. Starting at enrollment, index cases and contacts self-collected nasal specimens daily for 2 weeks — with day 0 defined as the day of symptom onset of the index case — and on days 17, 19, 21, and 28. Specimens were stored at –20°C in a designated freezer provided to the participants, collected weekly by study staff, and stored at –80°C long-term. Venous blood specimens were collected at enrollment and during weekly home visits at days 9, 14, 21, and 28 PSO of the index case. The timing in days PSO for each specimen was adjusted retrospectively for contact cases according to self-reporting. A survey was performed at or before enrollment to collect information on demographics, underlying conditions, prior infections, symptom start date, and vaccine doses received.

### Neutralizing antibody response assay.

The PhenoSense SARS CoV-2 nAb Assay (Monogram Biosciences) was used to determine NAb titers as described previously (45, 46). Briefly, the assay was done using HIV-1 pseudotype virions expressing SARS-CoV-2 spike proteins from Wuhan-Hu-1, Beta, P1, Epsilon, Delta, BA.1, and BA.2. Virions were generated in HEK293 cells (Monogram Biosciences) following cotransfection of a spike-encoding vector with an HIV-1 genomic vector expressing firefly luciferase. Reduction is luciferase activity in infected HEK293 cells expressing human Ace2 and TMPRSS2, following preincubation of pseudovirions with serial solutions of patient plasma, was used to determine the 50% infectious dose (IC_50_). NAb titers were determined at all available time points (days 7, 14, 21, and 28). Maximum NAb titer was defined as the NAb titer on the day with the highest NAb titers against the variant of interest, for each participant.

### RNA extraction.

RNA extraction from 200 μL of nasal specimens was done using the KingFisher (Thermo Fisher Scientific) automated extraction instrument and the MagMAX Viral/Pathogen Nucleic Acid Isolation Kit (Thermo Fisher Scientific) following the manufacturer’s instructions as previously described (8). For confirmatory quantitative PCR (qPCR) the Quick-DNA/RNA Viral MagBead kit (Zymo Research) was used as previously described (8).

### qPCR assay.

For each qPCR reaction, 4 μL of RNA sample were mixed with 5 μL 2× Luna Universal Probe One-Step Reaction Mix, 0.5 μL 20× WarmStart RT Enzyme Mix (New England Biolabs [NEB]), 0.5 μL of target gene specific forward and reverse primers, and probe mix as previously described (8). qPCR was run for SARS-CoV-2 nucleocapsid and envelope and for host mRNA RNaseP as a control for RNA extraction. In total, 8 μM each of forward and reverse primers and 4 μM probe for envelope; 5.6 μM each of forward and reverse primers and 1.4 μM probe for nucleocapsid; and 4 μM each of forward and reverse primers and 1 μM probe for RNaseP were used per reaction. Each 96 well qPCR plate was run with a 10-fold serial dilution of an equal mix of plasmids containing a full copy of nucleocapsid and envelope genes (Integrated DNA Technologies [IDT]), as an absolute standard for the calculation of RNA copies and primer efficiency assessment. qPCR were run on a CFX Connect Real-Time PCR detection system (Bio-Rad) with the following settings: 55°C for 10 minutes, 95°C for 1 minute, and then cycled 40 times at 95°C for 10 seconds followed by 60°C for 30 seconds. Probe fluorescence was measured at the end of each cycle. All probes, primers, and standards were purchased from IDT. A sample was considered to contain SARS-CoV-2 RNA if both nucleocapsid and envelope were detected at Ct < 40. To control for the quality of self-sampling, RNaseP Ct values 2 SDs from the mean of all samples were repeated or excluded. Maximum RNA viral load was defined as the RNA value on the day with the highest RNA viral load for each participant.

### Cytopathic effect (CPE) assay.

All anterior nares samples up to 14 days PSO of the index case were assayed for their ability to induce a CPE. In cases where CPE was still positive within days 11–14, we continued to test samples beyond day 14 until 3 consecutive samples were CPE negative. As described previously (8), CPE was assessed on Vero-hACE2-TMPRSS2 cells (gifted from A. Creanga and B. Graham Vaccine Research Center, NIAID, NIH). Cells were maintained at 37°C and 5% CO_2_ in DMEM (Thermo Fisher Scientific) supplemented with 10% FCS, 100 μg/mL penicillin/streptomycin (Thermo Fisher Scientific), and 10 μg/mL of puromycin (Thermo Fisher Scientific). In total, 200 μL of nasal specimen was added to a well of a 96-well plate and serially diluted 1:1 with DMEM supplemented with 1× penicillin/streptomycin over 2 additional wells. 100 μL of freshly trypsinized cells, resuspended in infection media (made as above but with 2Gibco Scientific penicillin/streptomycin, 5 μg/mL amphotericin B [BioWorld] and no puromycin) at 2.5 × 10^5^ cells/mL, were added to each sample dilution. Cells were cultured at 37°C and 5% CO_2_ and checked for CPE from day 2 to 5. After 5 days of incubation, the supernatant (200 μL) from 1 well from each dilution series was mixed 1:1 with 2× RNA/DNA Shield (Zymo Research) for viral inactivation and RNA extraction as described above. Among specimens with visible CPE, the presence of infectious SARS-CoV-2 was confirmed by qPCR using nucleocapsid primers as described above. Duration of infectious viral shedding was defined as days between symptom onset and the last day of CPE positivity for each participant. All assays were done in the BSL3 facility at Genentech Hall, UCSF, following the study protocol that had received Biosafety Use Authorization.

### Sequencing.

The ARTIC Network amplicon-based sequencing protocol for SARS-CoV-2 was used to sequence the nasal specimen with the highest copies of viral RNA per participant. Thawed RNA specimens were converted to cDNA using the Luna RT mix (NEB). Arctic multiplex PCR primer pools (IDT) (versions 4.1 and 5.3.2) were used to generate amplicons that were barcoded using the Native Barcode expansion kits 1–24 (Nanopore), pooled, and used for adaptor ligation. Libraries were run on a MinION sequencer (Oxford Nanopore Technologies) for 16 hours. Consensus sequences were generated using the nCoV-2019 novel coronavirus bioinformatics protocol using the MinIon Pipeline. Lineage determination was done using the online Pangolin COVID-19 Lineage Assigner.

### Statistics.

Categorical data were summarized as frequencies of the total population. Continuous data were summarized with median values and IQRs. Comparisons of group medians was done using 2-sided unpaired Wilcox rank sum tests. We assessed the relationship between NAb responses and viral shedding dynamics, stratified by variant and vaccination status, in unadjusted and adjusted analyses. Stratified by Delta versus Omicron infections, we used multivariable linear regression to assess the effect of baseline NAbs on viral outcomes (maximum RNA load, continuous variable; duration of infectious viral shedding, continuous), adjusting for age, time since vaccination (months), and Omicron subvariant. *P* values were used to determine the significance of statistical comparisons, and a value less than 0.05 was considered significant. Data were analyzed with custom scripts using R in RStudio (version 2023.06.1+524).

### Study approval.

This activity was reviewed by the UCSF IRB and by the Centers for Disease Control and Prevention, deemed not research, and was conducted consistent with applicable federal law and CDC policy (§See e.g., 45 C.F.R. part 46, 21 C.F.R. part 56; 42 U.S.C. §241(d); 5 U.S.C. §552a; 44 U.S.C. §3501 et seq). Written informed consent was received from all study participants.

### Data availability.

The datasets that support the findings are provided in the Supporting Data Values file, and additional deidentified data and analytic code can be requested for collaborative research from the corresponding author.

## Author contributions

MAGK, JDK, JNM, CMM, MJP, and RA designed the study and experiments. MAGK, MT, and AZ conducted clinical virology measurements and sequencing. MAGK, JDK, SL, SAG, GRA, SS, JNM, MBH, CMM, and RA carried out data analysis. JDK, SAG, JPR, KA, MCD, JYC, MFC, SP, AM, MS, AD, BV, ETR, DRM, BG, RLR, and SGD recruited the clinical cohort, conducted clinical follow up, and carried out specimen collections. AC, CP, and TW conducted NAb assays. MAGK, JDK, MBH, CMM, SS, MJP, and RA wrote the manuscript. All authors revised the manuscript and approved the final version for submission.

## Supplementary Material

Supplemental data

Supporting data values

## Figures and Tables

**Table 1:**
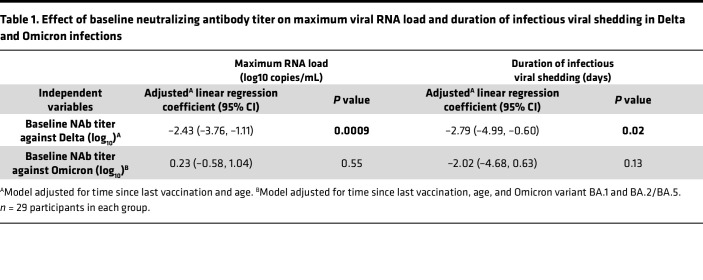
Effect of baseline neutralizing antibody titer on maximum viral RNA load and duration of infectious viral shedding in Delta and Omicron infections

## Version 1 03/10/2025

Electronic publication
