# Supplementary material for: Circulating neutralizing antibodies and SARS-CoV-2 variant replication following postvaccination infections
Source: JCI Insight. 2025 Mar 10;10(5):e185953. doi: 10.1172/jci.insight.185953 (PMC11949002; doi:10.1172/jci.insight.185953)
Supplement: Supplemental data [file jciinsight-10-185953-s306.pdf]

Supplemental Table 1. Participant characteristics (N=174).

**Characteristic**	**SARS-CoV-2-Infected**	**SARS-CoV-2-Uninfected**
**Total**	N=125	N=49
**Case**		
Index case[A], n (%)	78 (62.4)	0 (0.0)
Household contact (%)	47 (37.6)	49 (100.0)
**Sex, n (%)**		
Female	65 (52.0)	25 (51.0)
Male	60 (48.0)	24 (49.0)
**Age category, n (%)**		
13-17	3 (2.4)	3 (6.1)
18-29	27 (21.6)	11 (22.4)
30-39	43 (34.4)	13 (26.5)
40-49	28 (22.4)	12 (24.5)
50-59	11 (8.8)	7 (14.3)
>60	13 (10.4)	3 (6.1)
**Race/Ethnicity, n (%)**		
Hispanic or Latino Ethnicity	28 (22.4)	10 (20.4)
White	54 (43.2)	23 (46.9)
Black or African American	4 (3.2)	3 (6.1)
Asian	18 (14.4)	6 (12.2)
Pacific Islander/ Hawaiian	2 (1.6)	2 (4.1)
American Indian / Alaska Native	2 (1.6)	0 (0.0)
NA	17 (13.6)	5 (10.2)
**BMI, n (%)**		
<25	51 (40.8)	21 (43.0)
25 to 29.9	32 (25.6)	16 (32.7)
>30	25 (20.0)	6 (12.2)
NA	17 (13.6)	6 (12.2)
**COVID-19 vaccination status[B], n (%)**		
Unvaccinated	*57 (45.6)*	*21 (42.9)*
Primary series	*37 (29.6)*	*12 (24.5)*
Median (IQR) days since most recent dose	182 (123-229)	115 (89-150)
Most recent dose <180 days before baseline	23 (62.2)	12 (100)
Most recent dose 180+ days before baseline	14 (37.8)	0 (0)
One or two boosters	*31 (24.8)*	*15 (30.6)*
Median (IQR) days since most recent dose	178 (107-227)	109 (43-191)
Most recent dose <180 days before baseline	13 (41.9)	10 (66.7)
Most recent dose 180+ days before baseline	18 (58.1)	5 (33.3)

**Variant, n (%)**[C]		
Pre-VOC	45 (36.0)	NA
Epsilon	4 (3.2)	NA
Delta	34 (27.2)	NA
Omicron BA.1	10 (8.0)	NA
Omicron BA.2, BA.4 or BA.5	32 (25.6)	NA

[A]SARS-CoV-2 infected participant approached in each household

[B]Last vaccine dose 4 weeks or greater before study enrolment. 170/174 participants received either BNT162b2 (Pfizer) or mRNA-1273 (Moderna) primary vaccine series and when indicated, monovalent boosters. Two infected individuals received a Ad26.COV2.S (Janssen) primary series, one uninfected participant received a Ad26.COV2.S (Janssen) primary series and one uninfected participant a AZD1222 (AstraZeneca) primary series.

[C]Determined by sequencing of SARS-CoV-2 RNA from the participant or cohabiting participant's nasal specimen.

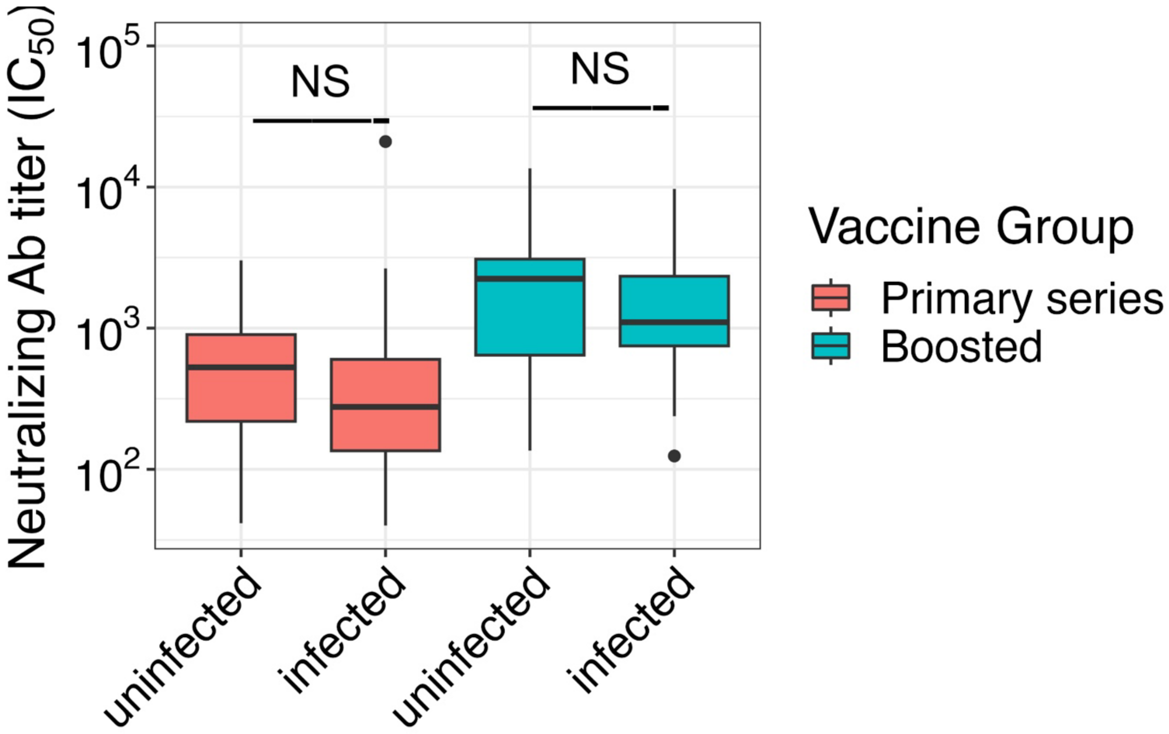

Supplemental Figure 1. NAb titers collected at recruitment in uninfected and infected participants. Comparison of NAb titers in recruitment specimens against Wuhan-Hu-1 in infected and uninfected participants stratified by vaccination status. Recruitment specimens were collected <7 days following symptom onset in infected individuals. Box plots represent the interquartile range (IQR; box), median (line), and IQR*1.5 values (whiskers). Statistical comparisons were done within vaccination groups by Wilcox test. NS, not significant.

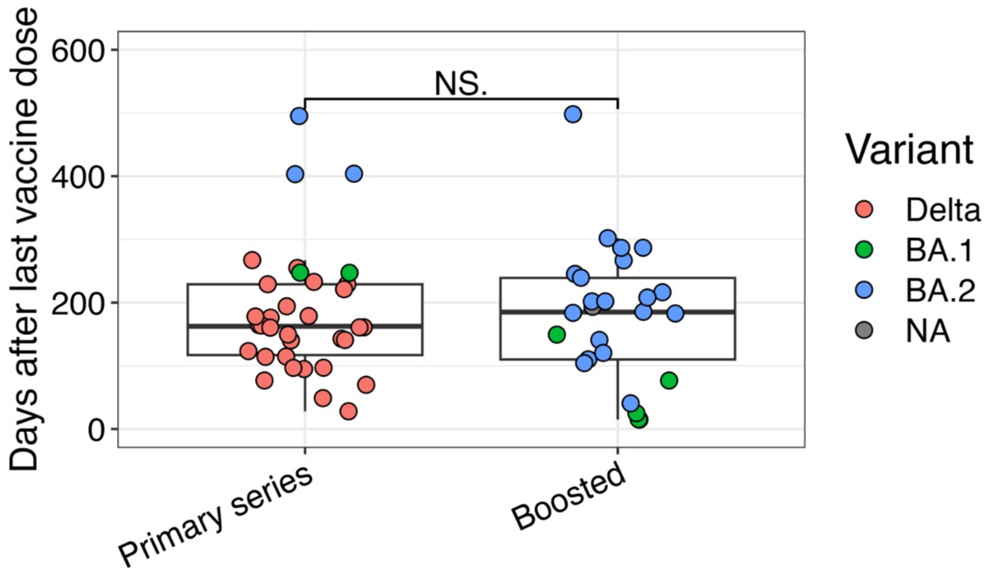

Supplemental Figure 2. Time since last vaccine dose in vaccinated participants. Comparison of the days since the last vaccine dose in participants who received a primary vaccine series or booster vaccinations. Box plots represent the interquartile range (IQR; box), median (line), and IQR*1.5 values (whiskers). Statistical comparison done by Wilcox test. NS, not significant.

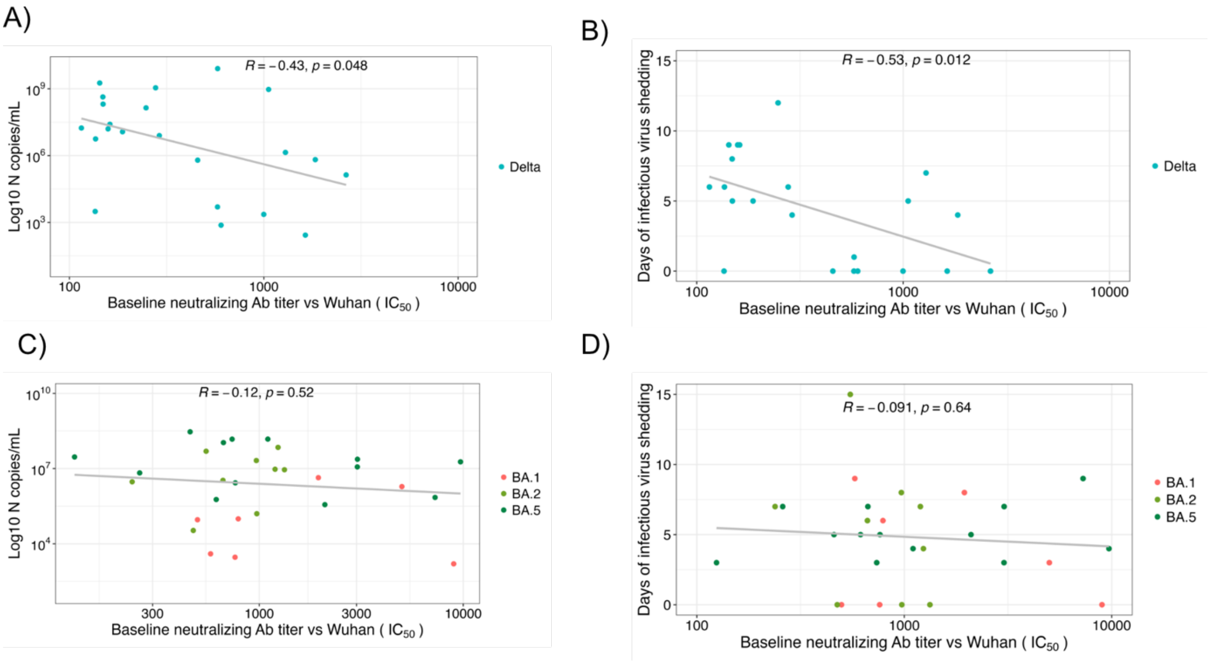

Supplemental Figure 3. The relationship between baseline NAb titers targeting Wuhan-Hu-1 and viral shedding outcomes. The correlation between baseline NAb titer targeting Wuhan-Hu-1 in vaccinated participants (N=29) infected with Delta variants and maximum RNA viral copies **(A)** and infectious virus shedding **(B)** over the study period. The correlation between baseline NAb titer targeting Wuhan-Hu-1 in vaccinated participants (N=29) infected with Omicron variants and maximum RNA viral copies **(C)** and infectious virus shedding **(D)** over the study period. Participants (N=9) with recruitment specimens taken on day 7 PSO or later were excluded. Pearson's correlation coefficients and infecting variant are shown.
